# Use of fomepizole as a novel approach for preventing aciclovir-induced neurotoxicity

**DOI:** 10.1093/jac/dkag313

**Published:** 2026-09-07

**Authors:** Åse Östholm, Erik Lindeman, Ylva Böttiger, Dan Eklund, Bertil Kågedal, Marie Studahl, Jan-Olov Höög, Håkan Hanberger, Anders Helldén

**Affiliations:** Clinical Department of Infectious Diseases in Östergötland, Region Östergötland, Linköping, Sweden; Department of Biomedical and Clinical Sciences, Linköping University, Linköping, Sweden; Swedish Poisons Information Centre, Stockholm, Sweden; Department of Biomedical and Clinical Sciences, Linköping University, Linköping, Sweden; Department of Clinical Pharmacology and Pharmacy Services, Region Östergötland, Linköping, Sweden; Clinical Department of Infectious Diseases in Östergötland, Region Östergötland, Linköping, Sweden; Department of Biomedical and Clinical Sciences, Linköping University, Linköping, Sweden; Department of Clinical Chemistry, Linköping University, Linköping, Sweden; Department of Infectious Diseases, Sahlgrenska University Hospital, Gothenburg, Region Västra Götaland, Sweden; Department of Infectious Diseases, Institute of Biomedicine, Sahlgrenska Academy, University of Gothenburg, Gothenburg, Sweden; Department of Medical Biochemistry and Biophysics, Karolinska Institutet, Stockholm, Sweden; Clinical Department of Infectious Diseases in Östergötland, Region Östergötland, Linköping, Sweden; Department of Biomedical and Clinical Sciences, Linköping University, Linköping, Sweden; Department of Biomedical and Clinical Sciences, Linköping University, Linköping, Sweden; Department of Laboratory Medicine, Division of Clinical Pharmacology, Karolinska Institutet, Karolinska University Hospital, Huddinge, Stockholm, Sweden

Aciclovir is the main treatment for varicella zoster virus (VZV) of the CNS.^1^ Although generally well tolerated, aciclovir is associated with nephrotoxicity^2^ and neurotoxicity,^3^ with the latter most strongly associated with accumulation of its metabolite 9-carboxymethoxymethylguanine (CMMG).^4^ Approximately 90% of aciclovir is excreted unchanged by the kidneys, whereas 8%–14% undergo metabolism by class I alcohol dehydrogenases (ADHs) to an aldehyde intermediate that is subsequently converted to CMMG by aldehyde dehydrogenase 2 (ALDH2) (Figure S1, available as Supplementary data at *JAC* Online).^5,6^ In renal failure, prolonged aciclovir exposure promotes sustained CMMG formation and accumulation. Serum CMMG concentrations > 10.8 µmol/L and CSF concentrations > 0.6 µmol/L strongly predict neurotoxicity and may help distinguish acyclovir-associated neurotoxicity from ongoing CNS infection.^4,7^

ADH inhibition could reduce CMMG formation by blocking the formation of the aldehyde intermediate. Fomepizole (4-methylpyrazole), a class I ADH inhibitor, is an approved therapeutic agent for treatment of methanol and ethylene glycol poisoning.^8^ We hypothesized that fomepizole could prevent CMMG formation and aciclovir-induced neurotoxicity, analogous to its prevention of toxicity in methanol and ethylene glycol poisoning.

An 80-year-old woman (51 kg, 157 cm) with no major comorbidities presented with dizziness, imbalance and progressive neurologic deficits. MRI on Day 5 showed inflammatory changes from the pontomedullary junction to the cervical spinal cord (Figure S2a and b). Intravenous aciclovir was initiated at 500 mg q8h based on a plasma creatinine of 70 µmol/L (Figure 1). CSF PCR testing was positive for VZV DNA. A single dose of ciprofloxacin 500 mg was administered on Day 7 due to suspicion of urinary infection. However, her neurological deficits persisted, and the dose was subsequently increased to 750 mg q8h at midnight on Day 8. That same morning, she was diagnosed with acute kidney injury and encephalopathy. Trough serum aciclovir and CMMG concentrations were elevated at 84 µmol/L and 64 µmol/L, respectively. Aciclovir was discontinued, and two intermittent haemodialysis (IHD) sessions were performed 1 day apart, resulting in rapid neurological recovery and decreased CMMG concentrations (Figure 1).

**Figure 1.**
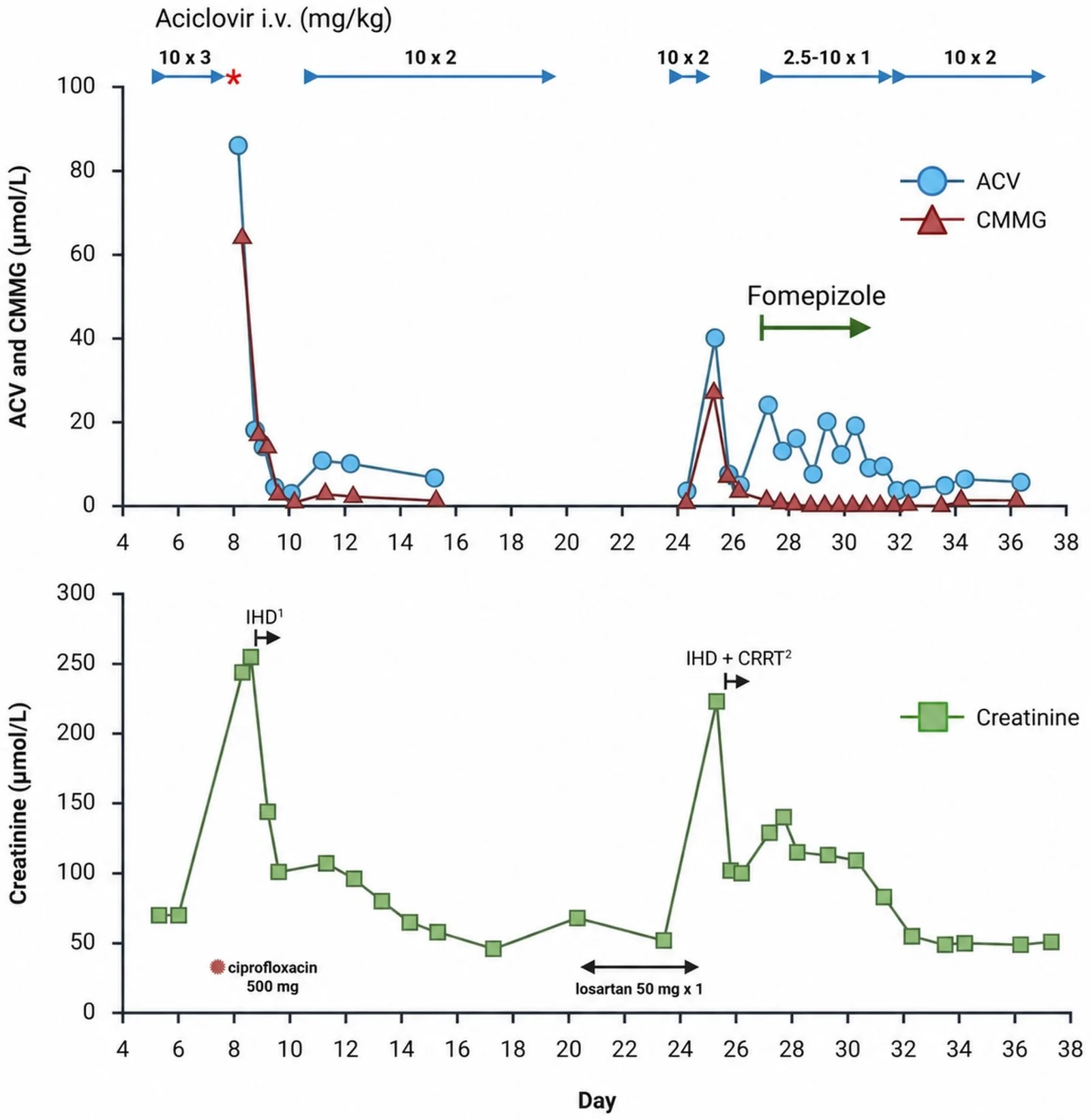
The patient’s serum aciclovir (ACV), 9-carboxymethoxymethylguanine (CMMG), and plasma creatinine concentrations during two episodes of aciclovir-associated neurotoxicity. Upper panel: ACV and CMMG concentrations. Lower panel: plasma creatinine concentrations. The aciclovir dosing schedule is shown at the top, and the red asterisk (*) indicates dose escalation to 15 mg/kg. Only trough aciclovir concentrations are shown; post-dose peak concentrations during fomepizole treatment ranged from 50 to 74 µmol/L. Black arrows indicate IHD and continuous renal replacement therapy (CRRT). Two 4-h IHD sessions were performed during the first episode. During the second period, a 4-h IHD session was performed, followed by 12 h of CRRT. The green arrow indicates the 4-day fomepizole treatment. Fomepizole therapy was initiated for 2 days according to the standard regimen for toxic alcohol poisoning (15 mg/kg loading dose followed by 10 mg/kg every 12 h). During the final two treatment days, after serum CMMG concentrations had fallen below the laboratory limit of detection, the dose was reduced to 5 mg/kg once daily and administered 30 min before the daily aciclovir dose. The timing of the single ciprofloxacin dose and initiation of losartan therapy is shown because both drugs may have contributed to the development of acute kidney injury. Created in BioRender. Lindeman, E. (2026) https://BioRender.com/y5rs22z.

As neurological symptoms recurred (Figure S3a), aciclovir was reinitiated on Day 10 at a reduced frequency of 500 mg q12h. Plasma creatinine concentrations and trough aciclovir and CMMG concentrations remained stable, and no neurotoxicity occurred. Aciclovir was discontinued on Day 20 after clinical and radiographic improvement, and losartan 50 mg daily was initiated for hypertension. However, recurrence of CNS lesions (Figure S3b) necessitated renewed aciclovir treatment 3 days later. Despite dose reduction, the patient again developed acute kidney injury and encephalopathy within 48 h, illustrating her marked susceptibility to recurrent aciclovir-associated toxicity. Haemodialysis resulted in prompt neurologic recovery (Figure 1). Given the need for continued antiviral therapy, aciclovir was resumed on Day 26 with dose adjustment guided by therapeutic drug monitoring and plasma creatinine. Fomepizole was co-administered with the intent to inhibit CMMG formation and prevent further neurotoxic reactions. Given the lack of previous clinical experience to guide fomepizole dosing for inhibition of aciclovir metabolism, the initial dosing schedule was based on the standard regimen used for ethylene glycol poisoning. The dose was subsequently reduced when serial measurements demonstrated a marked decrease in CMMG concentrations, consistent with inhibition of aciclovir metabolism. During fomepizole coadministration, serum CMMG concentrations remained below the performing laboratory’s limit of detection (<1 µmol/L) despite therapeutic aciclovir concentrations and persistent renal impairment (Figure 1). Notably, serum CMMG concentrations remained unmeasurable after reducing the fomepizole dose to 5 mg/kg once daily. No further neurotoxicity occurred, and no recurrent acute kidney injury was observed during fomepizole coadministration. After fomepizole was discontinued, CMMG concentrations increased to a maximum of 3 µmol/L. Follow-up imaging revealed improved CNS findings, and the patient was discharged on Day 64 on suppressive valaciclovir 250 mg twice daily without recurrence of VZV CNS infection or aciclovir-associated neurotoxicity.

The case findings align with the hypothesis that pharmacologic inhibition of aciclovir metabolism can suppress CMMG formation during aciclovir therapy.^9^ The decision to administer fomepizole was supported by prior *in vitro* experiments conducted in our laboratory (Figure S4) and by previously published pharmacogenetic evidence indicating that ALDH2 polymorphisms influence aciclovir clearance.^10^ The rapid resolution of neurological symptoms following haemodialysis during both toxicity episodes was typical in our experience and supports neurotoxicity rather than progression of VZV CNS infection as the cause of the clinical deterioration. Our case also illustrates the challenges of treating older patients with VZV CNS infection, who often have limited renal reserve and are vulnerable to acute kidney injury and aciclovir-associated neurotoxicity. In Sweden (population 10.5 million), the three laboratories analysing aciclovir and CMMG concentrations collectively diagnose 1–2 cases of aciclovir-induced neurotoxicity per week, illustrating that this complication is not uncommon. Although the role of fomepizole in preventing recurrent neurotoxicity during continued aciclovir therapy cannot be established from a single case, our findings suggest that adjunctive ADH inhibition may represent a strategy worthy of further investigation.

## Supplementary Material

dkag313_Supplementary_Data

## Supplementary data

Figures S1, S2, S3 and S4 are available as Supplementary data at *JAC* Online.
