# Supplementary material for: Use of fomepizole as a novel approach for preventing aciclovir-induced neurotoxicity
Source: J Antimicrob Chemother. 2026 Sep 7;81(10):dkag313. doi: 10.1093/jac/dkag313 (PMC13546786; doi:10.1093/jac/dkag313)
Supplement: Supplementary file 1 [file dkag313_supplementary_data.docx]

## Supplementary material

1. Figure S1: Figure showing the proposed metabolism of aciclovir.
2. Figure S2, A and B. Magnetic resonance imaging (MRI) of medullary edema, hospital day 5.
3. Figure S3, A and B. A) Axial T2FLAIR MRI hospital day 12. B) Axial T2-FLAIR MRI hospital day 23, showing new infarctions (white arrows).
4. Figure S4. Human liver cytosol was incubated with aciclovir and cofactors, demonstrating that fomepizole inhibits alcohol dehydrogenase and thereby reduces CMMG formation in vitro.

**Supplementary Figure S1.** Aciclovir is converted to an aldehyde intermediate by alcohol dehydrogenase (ADH), which is subsequently oxidised by aldehyde dehydrogenase 2 (ALDH2) to 9-carboxymethoxymethylguanine (CMMG), the principal metabolite of aciclovir. Fomepizole inhibits the initial step catalysed by class I ADH, preventing the formation of both the aldehyde and CMMG. A minor pathway is oxidation by aldehyde oxidase to 8-hydroxy aciclovir; however, this has not been quantified. Cofactors and reaction intermediates are shown. NAD^+^ = nicotinamide adenine dinucleotide.

*ALT TEXT Figure S1*: *Figure showing the proposed metabolism of aciclovir.*

**Supplementary Figure S2, A and B.** A) Coronal and B) axial T2-FLAIR magnetic resonance imaging (MRI), respectively, at the onset of myelitis symptoms on hospital day 5 showing medullary oedema (white arrows).

*ALT TEXT Figure S2, A and B. Magnetic resonance imaging (MRI) of medullary edema, hospital day 5.*

B

A

**Supplementary Figure S3, A and B.** A) Axial T2 FLAIR magnetic resonance imaging (MRI) on hospital day 12, two days after aciclovir 500 mg q12h was reinitiated. B) Axial T2-FLAIR MRI performed on hospital day 23 showed new infarctions in the pons (white arrows). Aciclovir therapy was therefore resumed on day 23 at a dose of 500 mg twice daily.

*ALT TEXT Figure S3, A and B.* *A) Axial T2FLAIR magnetic resonance imaging (MRI) hospital day 12. B) Axial T2-FLAIR MRI hospital day 23, showing new infarctions (white arrows).*

B

A

**Supplementary Figure S4.** Effect of the alcohol dehydrogenase inhibitor fomepizole (4-MP) on the *in vitro* metabolism of aciclovir to 9-carboxymethoxymethylguanine (CMMG). Human liver cytosol was incubated with aciclovir (100 µmol/L) in phosphate buffer (0.1 M, pH 7.8) containing nicotinamide adenine dinucleotide (NAD⁺; final concentration 2.4 mM) for 16 h at 37 °C, either without 4-MP or in the presence of 25 or 50 µmol/L 4-MP. The resulting CMMG concentrations were 1.88, 0.57, and 0.39 µmol/L, respectively. Thus, 25 and 50 µmol/L 4-MP reduced CMMG formation by approximately 70% and 79%, respectively. The assay was not conducted in replicates.

***ALT TEXT*** *Figure S4***.** *Human liver cytosol was incubated with aciclovir and cofactors, demonstrating that fomepizole inhibits alcohol dehydrogenase and thereby reduces CMMG formation in vitro.*
